# 《肺癌骨转移诊疗专家共识》审稿会会议纪要

**DOI:** 10.3779/j.issn.1009-3419.2013.11.07

**Published:** 2013-11-20

**Authors:** 修益 支

**Affiliations:** 100053 北京，首都医科大学肺癌诊疗中心，首都医科大学宣武医院胸外科 Beijing Lung Cancer Center, Department of Toracic Surgery, Beijing Xuanwu Hospital, Capital Medical University, Beijing 100053, China

肺癌是我国发病率和病死率最高的恶性肿瘤。随着治疗手段的进步和靶向药物的应用，晚期肺癌患者的中位生存时间逐渐延长，骨转移及骨相关事件的发生率亦随之增高。因此，肺癌骨转移的规范化治疗，受到越来越多临床医生的重视。

由中国癌症基金会、中华医学会胸心血管外科学会肺癌学组、中国抗癌协会肺癌专业委员会、中国抗癌协会化疗专业委员会共同牵头举办的《肺癌骨转移诊疗专家共识》第一次审稿会议于2013年8月23日在北京召开。会议由中国癌症基金会副主席孙燕院士和国家卫计委《原发性肺癌诊疗规范》专家组组长、中华胸心外科学会肺癌学组组长支修益教授主持，中国抗癌协会肺癌专业委员会主任委员王长利教授、中国抗癌协会化疗专业委员会主任委员石远凯教授和国内肺癌临床诊疗领域的十余位知名专家出席了会议。与会专家就《肺癌骨转移诊疗专家共识》（草案）中的五大议题展开热烈的讨论，分别涉及：肺癌流行病学、肺癌骨转移发病机制和临床诊疗流程、肺癌骨转移药物治疗、外科手术和放射治疗推荐，以及癌性骨痛治疗等。分享了目前肺癌骨转转移临床诊疗领域的最新成果，讨论制定了指导肺癌骨转移临床规范化治疗的各项诊疗细则。

在诊断方面，针对各项临床影像和实验室检测方法，如ECT、CT、MR、X线和组织学检查的敏感性和特异性，结合临床实践，借鉴国外相关诊疗规范，制定了具有可操作性的肺癌骨转移临床路径和诊断流程图。在治疗方面，深入探讨了各种肺癌骨转移治疗方法的治疗原则、适应症和具体方案等。着重讨论了目前各种主要抗癌治疗方法的适应症：如化疗适合于多发骨转移灶、化学敏感肿痛所致的神经伤害性或神经病理性疼痛综合征；放疗适合于孤立骨转移灶（体外放射治疗）、多发骨转移灶（放射性核素治疗）、脊髓外压迫、外周神经肿瘤性压迫或侵犯所致疼痛或功能障碍；骨外科手术适合于病理性骨折及脊髓压迫固定术等；并强调了双膦酸盐在肺癌骨转移治疗中基础用药的地位，以及伊班膦酸良好的安全性，是唯一可以使用负荷疗法快速缓解骨痛的双膦酸盐。并就此制定了肺癌骨转移多学科综合治疗流程图，为临床医生提供了详细的诊疗流程。本共识的另一个亮点，增加了肺癌骨转移的心理治疗模块，强调晚期肺癌患者的身-心-并重的人性化治疗，符合生物-心理-社会医学模式，使晚期肺癌患者最大获益。

支修益教授在会议总结中提出：肺癌骨转移治疗策略，是在规范化个体化治疗基础上的多学科综合治疗，是从一个新的高度来认识肺癌骨转移的诊治模式。多学科综合治疗的概念，是局部治疗与全身治疗的结合，是多学科协作的综合治疗，避免以往各学科科室各自为战，片面治疗的不规范现象。一个整体化的治疗策略，能更好地缓解晚期肺癌患者的癌痛症状、有效预防和正确处理骨相关事件、切实提高患者生存质量，并延长晚期肺癌患者生命。

经过专家们的热烈讨论，与会专家在《肺癌骨转移诊疗专家共识》（讨论稿）框架和相关章节内容方面达成共识。同时，专家们提出还需要提供更多的循证医学证据，与会专家将分别代表各自的专业学术领域，进一步完善相关学科领域的学术内容。我们相信《肺癌骨转移诊疗专家共识》（2014年版）的出台，将会帮助临床医生进一步规范肺癌骨转移诊疗行为，并为广大晚期肺癌患者提供更有利的治疗方案。期待着《肺癌骨转移诊疗专家共识》（2014年版）早日问世临床。

**Table:** 附：肺癌骨转移诊疗专家组名单

**专家组顾问：**		
**孙燕**	**院士**	**中国医科院肿瘤医院**
**管忠震**	**教授**	**中山大学附属肿瘤医院**
**廖美玲**	**教授**	**复旦大学附属胸科医院**
**专家组组长：**		
**支修益**	**教授**	**首都医科大学宣武医院**
**石远凯**	**教授**	**中国医科院肿瘤医院**
**王长利**	**教授**	**天津医科大学肿瘤医院**
**编委会成员名单：（按照姓氏笔画顺序排列）**		
**于欣**	**教授**	**北京大学精神卫生研究所**
**王洁**	**教授**	**北京大学肿瘤医院**
**牛晓辉**	**教授**	**北京积水潭医院**
**刘云鹏**	**教授**	**中国医科大学附属第一医院**
**刘孟忠**	**教授**	**中山大学附属肿瘤医院**
**张沂平**	**教授**	**浙江省肿瘤医院**
**杨跃**	**教授**	**北京大学肿瘤医院**
**沈靖南**	**教授**	**中山大学附属第一医院**
**陈公琰**	**教授**	**黑龙江省肿瘤医院**
**周清华**	**教授**	**天津医科大学**
**周彩存**	**教授**	**上海肺科医院**
**郭其森**	**教授**	**山东省肿瘤医院**
**梁军**	**教授**	**青岛大学附属医院**
**程颖**	**教授**	**吉林省肿瘤医院**

